# Intestinal helminth co-infection and associated factors among pulmonary tuberculosis patients in Africa and Asia: a systematic review and meta-analysis

**DOI:** 10.1186/s12879-023-08716-9

**Published:** 2023-10-30

**Authors:** Yohannes Zenebe, Meseret Habtamu, Markos Abebe, Begna Tulu, Abay Atnafu, Daniel Mekonnen, Roland Lang, Abaineh Munshea

**Affiliations:** 1 Department of Medical Laboratory Science, College of Medicine and Health Sciences, Bahir Dar University, Bahir Dar, Ethiopia; 2 Health Biotechnology Division, Institute of Biotechnology, Bahir Dar University, Bahir Dar, Ethiopia; 3 Armauer Hansen Research Institute, Addis Ababa, Ethiopia; 4 Institute for Clinical Microbiology, Immunology and Hygiene, University Hospital of Erlangen, Friedrich-Alexander University, Erlangen-Nuremberg, Germany; 5 Department of Biology, Science College, Bahir Dar University, Bahir Dar, Ethiopia

**Keywords:** Tuberculosis, Intestinal helminths, Co-infection, Pooled prevalence, Africa, Asia

## Abstract

**Introduction:**

Tuberculosis (TB) and intestinal helminths have huge public health importance, and they are geographically overlapped. Data about the burden of intestinal helminth and TB co-infection in these areas are fragmented. In this systematic review and meta-analysis we compile the current literatures and generate pooled prevalence. We also identity factors associated with intestinal helminth co-infection among TB patients.

**Methods:**

Original articles published in English language up to March 23, 2022 were systematically searched from electronic database (PubMed/Medline, Scopus, Science Direct, Google Scholars and HINARI). The search was done using medical subject heading terms and keywords. Identified articles were exported into the EndNote library. The identified articles were screened using PRISMA flow diagram. Then the methodological quality of included articles was evaluated and rated using the modified version of Newcastle–Ottawa Scale. Data were extracted using Microsoft Excel. Sensitivity analysis and Egger regression test were used for the assessment of heterogeneity and publication bias. Finally the results are presented with a meta-analysis of pooled estimates, forest plots, and tables. The quantitative data were analyzed using Stata version 14.

**Results:**

From a total of 5457 searched articles, 22 eligible articles were included in the review. The pooled prevalence of helminth co-infection among TB cases was 29.69% (95%CI: 21.10, 38.29). TB patients were found to more frequently harbor one or more intestinal helminths than TB negative individuals (OR = 1.72 (95%CI: 1.20, 2.48))*.* Among the reported helminths, *Schistosoma mansoni* and *Strongyloides stercoralis* had the highest pooled prevalence among TB cases. However, unlike other individual helminths, only *Strongyloides stercoralis* (OR = 2.67 (95% CI, 1.20–6.76)) had significant association with TB cases compared to TB negatives. BMI was significantly associated with intestinal helminth co-infection among TB patients (OR = 2.75 (95%CI: 1.19, 6.38)).

**Conclusions:**

Patients with TB have been shown to harbor co-infection with one or more intestinal helminths with considerable proportions when compared with TB-negative individuals. The higher prevalence of helminth infection in TB cases might indicate that co-infection promotes active TB disease. Thus, routine intestinal helminth screening and assessment of their nutritional status is suggested for TB patients.

**Supplementary Information:**

The online version contains supplementary material available at 10.1186/s12879-023-08716-9.

## Introduction

Tuberculosis (TB) and intestinal helminth infections are geographically co-existing health problems and the most prevalent infectious diseases, mainly in middle and low-income countries [1]. Helminth infection is an area which remains largely ignored. Globally, more than 2 billion people are infected with intestinal helminths, and particularly soil transmitted helminths (STHs) affect more than 1.5 billion people in Africa, Asia and Latin America [2]. About one third of the people worldwide are also infected with *Mycobacterium (M.) tuberculosis* [1]. Africa and South-East Asia account for more than 70% of the global TB burden [3]. The geographical overlap of TB and other debilitating infectious diseases, such as intestinal helminths and HIV, makes the situation worse than in other WHO regions [4]. Moreover, intestinal helminth infection and TB are considered as poverty related diseases. Hence, the co-infection of intestinal helminths and *M. tuberculosis* is common, especially in African and Asian countries, where the burden of poverty related factors is high [3, 5, 6]. Up on this information, our review is specifically targeted in Africa and Asia to compile and analyze available evidences related to *M. tuberculosis* and intestinal helminths co-infection.

Co-infection from helminth and *M. tuberculosis* is not surprising in geographical areas where both diseases are frequent. The two infectious pathogens use several self-governing mechanisms to heighten susceptibility of the host and impact on their infection outcomes [7, 8]. Thus, it is conceivable that both infections can mutually change the susceptibility to and the course of disease.

Different individual studies showed significant correlation between intestinal helminth and TB [9–12]. Other studies did not find an association [13, 14]. However, a review of 11 studies done in a single country (Ethiopia) showed higher pooled prevalence of intestinal parasite infection among TB cases [15]. One more single review of 20 studies at the global scale, reported by Taghipour et al., also showed considerable pooled prevalence of intestinal helminth infection [16]. The above two reviews indicated substantial rate of helminth infection among TB cases. However, many questions remain to be answered, such as regional subgroup analysis for differences between geographic regions, or the role of possible risk factors associated with co-infection. In addition, the study of co-infections with helminths and TB is an active research field requiring intermittent analyses of the current state of the literature to obtain up-to-date information, especially in regions of low/middle-income countries.

Hence, this systematic review and meta-analysis is aimed to generate up-to-date information about the burden of intestinal helminth and *M. tuberculosis* co-infections in African and Asian countries. Moreover, the subgroup analysis of intestinal helminth co-infection across different regions/countries and assessment of possible risk factors may give a more detailed picture to comprehend the effect of helminths infection on active TB development or the effect in vice-versa.

## Materials and methods

### Protocol

The protocol of this review was developed based on the Preferred Reporting Items for Systematic Reviews and Meta-Analyses (PRISMA) reporting checklist and registered at Prospective Register of Systematic Reviews (PROSPERO) ID: CRD42022315731.

### Eligibility criteria

Original articles (prospective and retrospective cross-sectional, case–control, and cohort studies), which are reported on TB and intestinal helminths co-infection in different countries of Africa and Asia, and written in English language were included. However, review papers, conference papers, editorials, commentaries, case reports/case series, and articles published out of the study population and English language were excluded. Studies were reviewed based on the criteria of PICOS (participants, interventions, comparison, outcome, and study setting).

### Search strategy

Original articles published in English up to March/2022 were systematically searched from electronic databases such as, PubMed, Scopus, Science Direct, Google Scholars and HINARI. Grey literatures were retrieved from university databases and article preprint sources, medRxiv and bioRxiv. The reference lists of related reviews were screened to identify additional articles. The article search was done using medical subject headings (MeSH) terms and keywords with an appropriate combination using Boolean operators “AND” and “OR”. The search algorithm was (((((((("Helminths"[Mesh]) OR "Helminthiasis"[Mesh]) OR "Intestinal Diseases, Parasitic"[Mesh]) AND "Mycobacterium tuberculosis"[Mesh]) OR "Tuberculosis, Pulmonary"[Mesh]) OR "Tuberculosis"[Mesh]) AND "Africa"[Mesh]) OR "Asia"[Mesh]) AND "Coinfection"[Mesh]. In addition we used other related search algorisms to access more articles.

### Study selection and data collection process

All of the identified articles were exported into the EndNote library. After removing the duplicates, articles identified as potentially relevant by screening of title and summary were further evaluated by reading the full paper. Article selection was done following the PRISMA flow chart. Papers which were not eligible for the review were excluded and the reason for exclusion is documented in the flow chart. Data extractions were carried out after piloting the data extraction sheet. Data were extracted by two of the authors (YZ, DM) independently and any discrepancies on the data item were resolved by discussion and 3^rd^ party judgment (BT).

### Data items

After the selection of eligible articles, the findings of the papers were extracted using a data extraction template. The data extraction included, the name of the first author, year of publication, study area/country, study design, sample size, mean age, TB screening method, intestinal helminth screening method, and the number and types of intestinal helminths. Subgroup data containing outcomes of intestinal helminths among TB patients and TB negative participants, multiple helminthic infections, sex, age, residence, educational status, HIV status of TB patients, and BMI were extracted in Microsoft excel sheet.

### Condition being studied

The domains being studied in this review were the pooled prevalence, associated factors, and subgroup analysis of intestinal helminth co-infection among TB patients in African and Asian countries.

### Participant/population

In this review the target population was active TB patients with the comparator of TB negative individuals. Articles we included were observational study types, without any intervention on their study participants. The pooled prevalence of helminth-TB co-infection was the proposed outcome variable for this review.

### Context

We included articles reporting results of community or facilities-based studies which have been conducted in either Africa or Asia.

### Definitions

#### TB positives (cases)

Pulmonary TB presumptive participants who were microscopic or culture or Xpert MTB/RIF positive for *M. tuberculosis.*

#### TB negatives (controls)

Pulmonary TB presumptive participants, who were microscopy or culture or Xpert MTB/RIF negative for *M. tuberculosis.*

### Quality assessment

The methodological qualities of included articles were appraised in duplicate (YZ and DM). For the quality assessment, the modified version of Newcastle–Ottawa Scale (NOS) was used [17]. The NOS includes 3 categorical criteria with a maximum score of 9 points. The quality of each study was rated using the following scoring algorithms: points of ≥ 7, 3 to 6 and < 3 were considered as “good”, “fair”, and “poor” quality studies, respectively. Therefore, in order to improve the validity of this systematic review result, we only included primary studies with fair and good quality [18].

### Summary measures and risk of bias in individual studies

The data extracted from included papers were entered into Microsoft Excel and analyzed by Stata 14 software. Sensitivity analysis and Egger regression test were used for the assessment of heterogeneity and publication bias. The I^2^ statistic value of 25%, 50%, and 75% were used to declare the heterogeneity test as a low, medium, and high, respectively. In case of significant heterogeneity, a random effect model was used for the analysis [19]. The fixed model effect was also used for the analysis of non-significant heterogeneity. Publication bias was explored using visual inspection of the funnel plot.

### Synthesis of result

The collected data were analyzed using qualitative and quantitative measures via Stata 14 statistical software. We then computed the effect size (ES) and odds ratio (OR) for the analysis of the pooled prevalence and determinant factors of TB-helminth co-infections, respectively. Forest plot was used to assess the effect of risk factors. From the forest plots described in this review, the box indicated weight of articles from random effect analysis. The crossed line is the 95% confidence interval (CI); the solid vertical line is zero to x-axis.

### Subgroup analysis

We carried out a subgroup analysis and meta-regression of heterogeneous papers, with the relationship between helminthic infection and TB, according to the region of the study conducted.

## Results

### Study characteristics

**Fig. 1:**
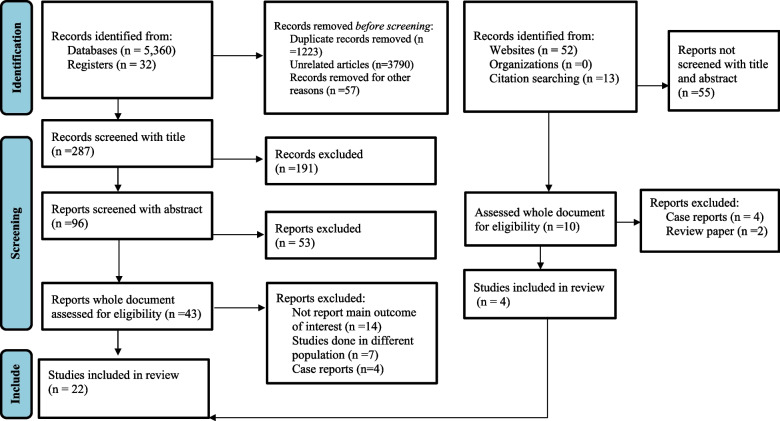
Flow diagram shows the included studies for the systematic review and meta-analysis of intestinal helminth co-infection among TB patients

A total of 5457 research articles were explored from different scientific data bases, registers and other sources, as explained from the methodology part (S2 Table). Among these, 5105 papers were removed due to duplication, irrelevance to our objective and other reasons. Only 352 research articles were screened by their title and abstract. Finally, 53 research articles were selected for full paper evaluation. After intensive screening, we found 22 studies [4, 9–12, 20–36] as eligible articles for final analysis (Fig. 1). From the included articles the number of case control, cohort and cross-sectional studies were 6, 1 and 15, respectively (Table 1). Among the 22 eligible articles, 27% of them used microscopic and kato-katz techniques, 63% used microscopic and concentration techniques, and 9% used microscopic, concentration, PCR and serology methods for helminth diagnosis. The total number of participants among TB cases was 4,986 and those of the control group 3,246 with a sample size ranging from 16 to 668 (S1 file). The mean age of participants among TB cases and TB negatives (controls), were 35.27 ± 7.30 years and 35.75 ± 8.71 years, respectively.

**Table 1:** Summary of research articles included in the systematic review and meta-analysis (*n* = 22)

No	Authors and years of publication (reference No.)	Study area	Study design	Sample size		Helminth prevalence among TB cases P (95% CI)	Helminth prevalence among controls P (95% CI)	Quality assessment (S3 Table)
				TB cases	TB free			
1	Abate et al., 2012 [12]	Ethiopia	Case control	112	112	28.57 (20.20, 36.94)	20.54 (13.05, 28.02)	7 points
2	Alemu et al., 2017 [22]	Ethiopia	Cross sectional	213	–-	24.41 (18.64, 30.18)	––––-	6 points
3	Mhimbira et al., 2017 [29]	Tanzania	Cohort study	597	375	31.83 (28.09, 35.56)	25.87 (21.43, 30.30)	7 points
4	Abate et al., 2015 [20]	Ethiopia	Case control	424	–-	36.79 (32.20, 41.38)	––––-	5 points
5	Alemayehu et al.,2014 [21]	Ethiopia	Cross sectional	72	343	29.17 (18.67, 39.67)	18.95 (14.80, 23.10)	4 points
6	Alemu et al.,2019 [11]	Ethiopia	Cross sectional	91	89	10.99 (4.56,17.41)	2.25 (-0.83, 5.33)	6 points
7	Elias et al.,2006 [4]	Ethiopia	Case control	230	510	70.87 (65.00, 76.74)	36.27 (32.10, 40.45)	5 points
8	Gashaw et al.,2019 [23]	Ethiopia	Cross sectional	259	––	10.04 (6.38, 13.70)	––––-	5 points
9	Hailu et al.,2015 [24]	Ethiopia	Case control	100	168	29.00 (20.11, 37.89)	7.74 (3.70,11.78)	6 points
10	Kassu et al.,2007 [25]	Ethiopia	Cross sectional	257	––	44.36 (38.28, 50.43)	–––––-	5 points
11	Lemma et al.,2022 [27]	Ethiopia	Cross sectional	350	––	10.00 (6.86, 13.14)	–––––-	5 points
12	McLaughlin et al.,2021 [9]	Kenya	Cross sectional	194	315	25.26 ((19.14,31.37)	30.79 (25.70, 35.89)	7 points
13	Range et al.,2007 [30]	Tanzania	Cross sectional	532	123	56.39 (52.18, 60.60)	42.28 (33.55, 51.01)	5 points
14	Sikalengo et al.,2018 [31]	Tanzania	Cross sectional	668	––	23.05 (19.86, 26.25)	–––––	7 points
15	Tegegne, et al.,2018 [33]	Ethiopia	Cross sectional	43	213	11.63 (2.05, 21.21)	14.55 (9.82, 19.29)	4 points
16	Tesfaye, et al.,2022 [34]	Ethiopia	Cross sectional	36	206	8.33 (-0.70, 17.36)	4.85 (1.92, 7.79)	5 points
17	Kusumaningtyas et al.,2020 [26]	Indonesia	Cross sectional	16	––	56.25 (31.94, 80.56)		4 points
18	Li, X. X. et al.,2014 [28]	China	Cross sectional	369	366	7.59 (4.89, 10.29)	8.20 (5.39, 11.01)	7 points
19	Panigrahi et al.,2019 [10]	India	Cross sectional	84	34	30.95 (21.07, 40.84)	5.88 (-12.33, 29.53)	5 points
20	Wong et al.,2019 [35]	Malaysia	Cross sectional	82	55	85.37 (77.72, 93.02)	89.09 (80.85, 97.33)	4 points
21	Taghipour et al. 2019 [32]	Iran	Case control	161	181	1.86 (-0.23, 3.95)	0.55 (-0.53, 1.63)	6 points
22	Okodua et al. 2010 [36]	Nigeria	Case control	96	156	28.13 (19.13, 37.12)	21.79 (15.32, 28.27)	7 points

*N.B: The numbers in bracket, in front of authors, are the reference numbers of each article included in the analysis*

**Fig. 2:**
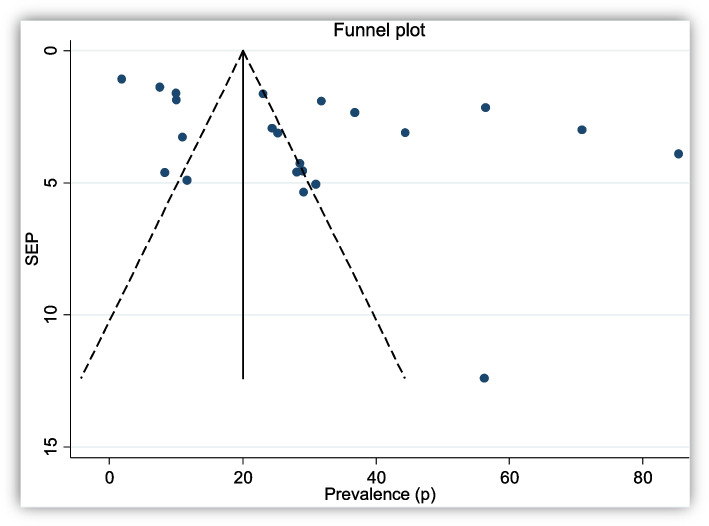
Funnel plot for pooled prevalence of intestinal helminth co-infection among TB patients in Africa and Asia. The Y-axis and X-axis shows the standard error of prevalence (SEP) and prevalence (P) distribution of each article included in this review, respectively. The dots indicate the prevalence of each article and they are scattered asymmetrically; which shows the heterogeneity of included articles

The publication bias was measured using a funnel plot and Egger’s test. Egger’s test showed a publication bias (*P* = 0.016). The funnel plot was also more or less asymmetrical; that shows the heterogeneity of included articles (Fig. 2).

### Prevalence of intestinal helminth co-infection among TB patients and controls

**Fig. 3:**
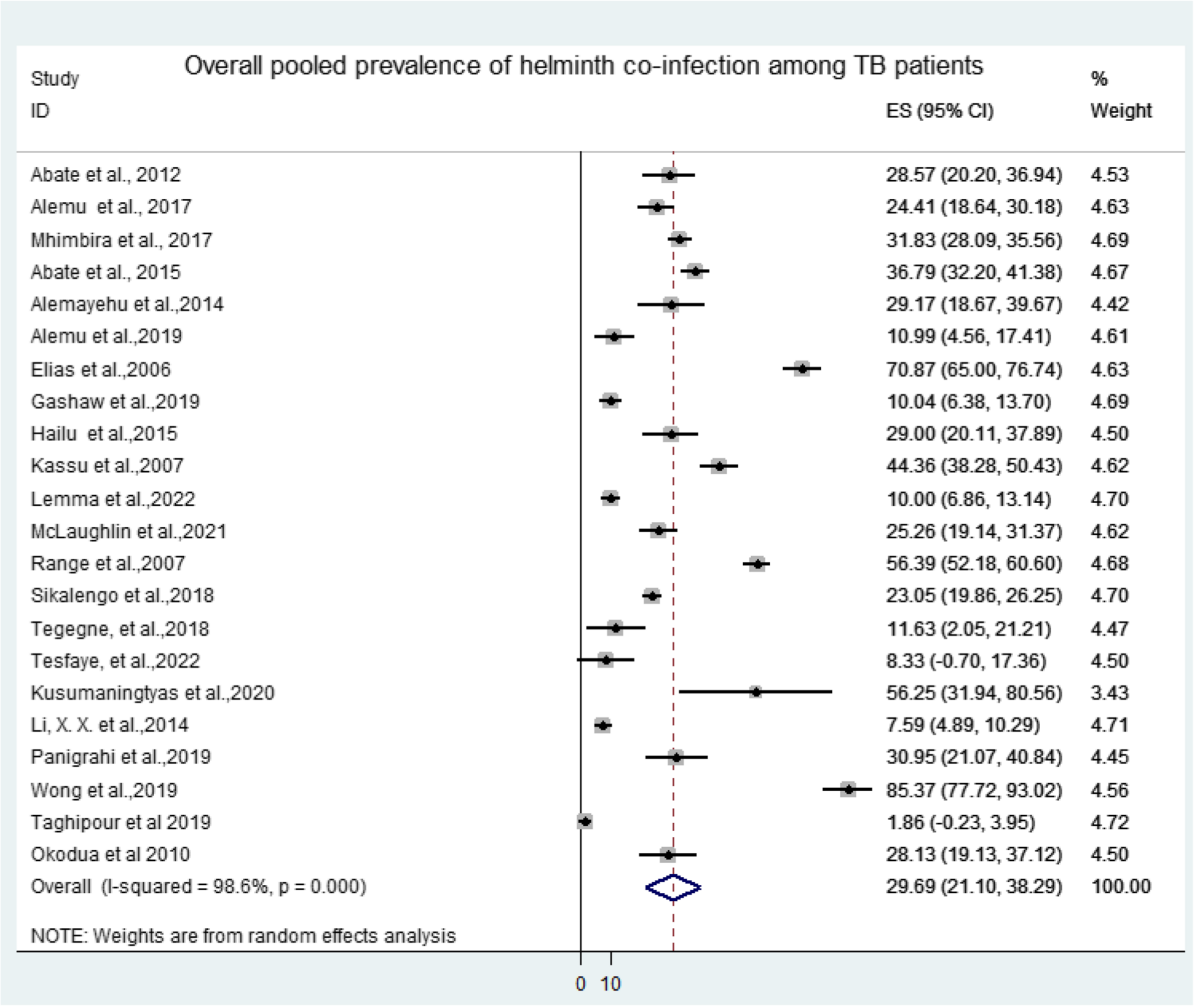
Forest plot of overall intestinal helminth pooled prevalence among TB cases in Africa and Asia: In the forest plot, the box indicated weight of articles from random effect analysis. The horizontal lines show the 95% confidence interval (CI); the solid vertical line is zero to x-axis

**Fig. 4:**
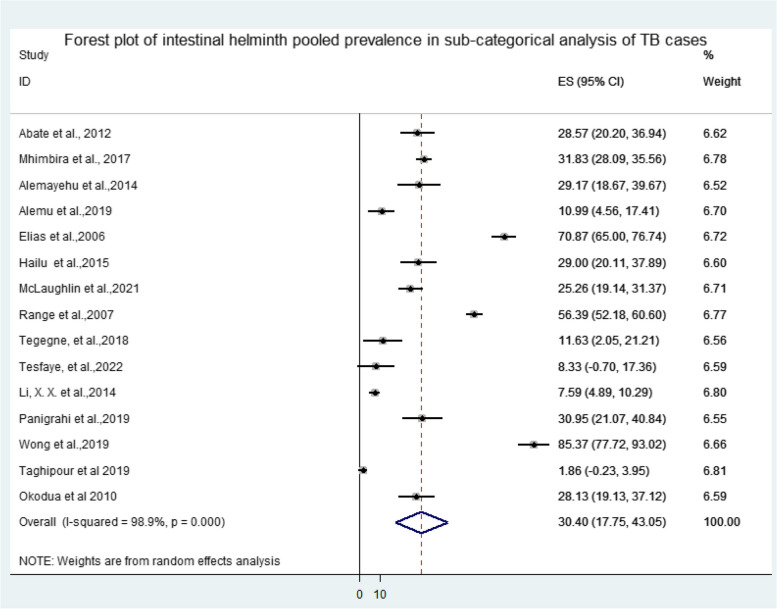
Forest plot of intestinal helminth pooled prevalence in sub-categorical analysis of TB cases

**Fig. 5:**
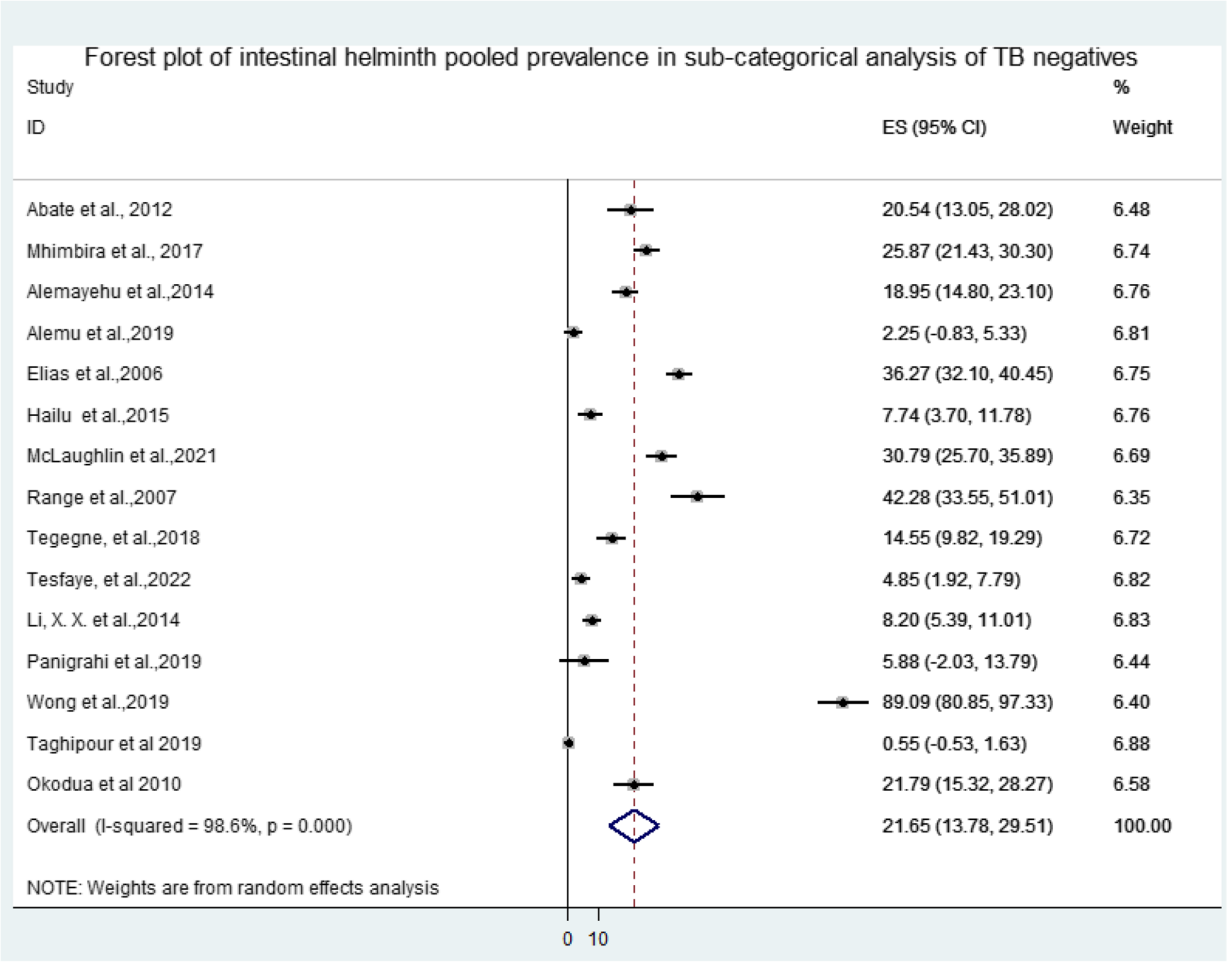
Forest plot of intestinal helminth pooled prevalence in sub-categorical analysis of TB negatives

The overall pooled prevalence of intestinal helminth infection among TB patients reported from 22 selected articles was 29.69% ((95%CI: 21.10, 38.29), *P* < 0.001, I^2^ = 98.6%) (Fig. 3). Among the selected articles, 15 reported both intestinal helminth infection among TB cases and TB negative participants. Thus, the pooled prevalence of intestinal helminth infection among TB cases and controls were 30.40% ((95%CI: 17.75, 43.05), *P* < 0.001, I^2^ = 98.9%) and 21.65% ((95%CI: 13.78, 29.51), *P* < 0.001, I^2^ = 98.6%), respectively (Figs. 4 and 5). A wider range in the prevalence of intestinal helminth co-infection among TB patients (1.86% in Iran and 85.37% in Malaysia) and TB negatives was observed (0.55% in Iran and 89.09% in Malaysia) (Fig. 3) (S1 file).

### Subgroup analysis of intestinal helminth prevalence

The subgroup analysis of intestinal helminth prevalence among TB cases by region indicated that higher pooled prevalence in Asia, 35.30%, (95%CI: 13.98, 56.63) than in Africa, 28.24%, (95%CI: 19.46, 37.02). Further subgroup analysis showed highest intestinal helminth pooled prevalence in other African countries, 33.02%, (95%CI: 19.97, 46.06) than in Ethiopia, 26.22% (95%CI: 15.20, 37.24) (S2 file).

### Associations of intestinal helminth infection and tuberculosis

**Fig. 6:**
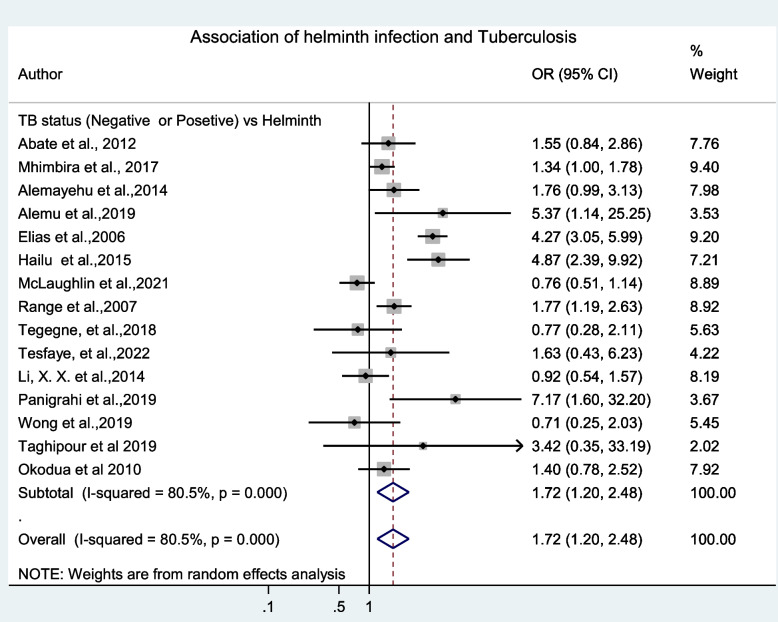
Forest plot of odds ratio for the association of intestinal helminth and *M. tuberculosis* infections

From this systematic review and meta-analysis, sub-categorical analysis was done. To assess the association of intestinal helminth infection with TB, 15 research articles (reported helminth status for both TB negative and TB positive participants), were included for analysis [4, 9–12, 21, 24, 28–30, 32–36]. The results indicated that TB cases were more prone to than TB negative participants for being co-infected with intestinal helminths, (OR = 1.72 (95%CI: 1.20, 2.48), *p* < 0.001, I^2^ = 80.5%) (Fig. 6).

### Risk factors of intestinal helminth infection among TB cases

**Fig. 7:**
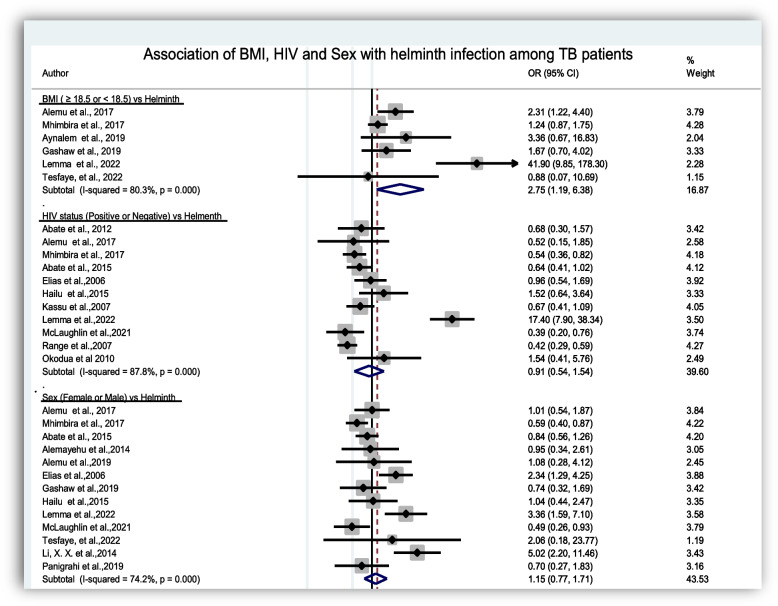
Forest plot of odds ratio for the association of BMI, HIV and Sex for intestinal helminth infection of TB cases

**Fig. 8:**
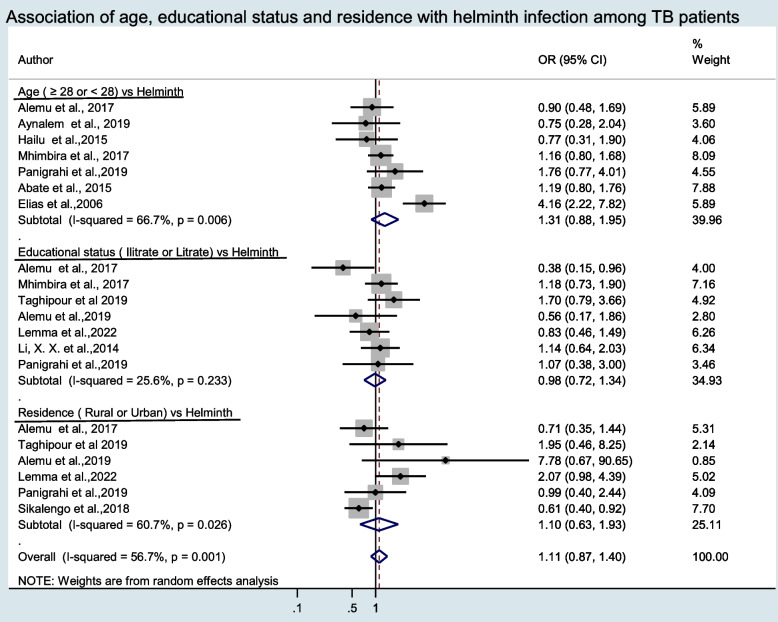
Forest plot of odds ratio for the association of age (year), educational status and residence for intestinal helminth infection of TB cases

In this systematic review and meta-analysis risk factors such as HIV infection, body mass index (BMI), sex, age, educational status, and residence were analyzed (Figs. 7 and 8). Thirteen articles among TB cases and six articles among controls reported about HIV status. The pooled prevalence of HIV among TB cases and controls were 30.29%, (95%CI: 22.18, 38.39) and 24.66%, (95%CI: 12.16, 37.16), respectively. The pooled meta-regression analysis showed a statistically significant association between low BMI and intestinal helminth infection in TB cases, with an OR of 2.75, (95%CI: 1.19, 6.38). However, the above remaining variables did not show statistically significant association with intestinal helminth infection among TB cases (Figs. 7 and 8).

### Pooled prevalence of different intestinal helminth species

The included research articles reported eight types of intestinal helminths. Under TB patient category, 19 papers [4, 9–12, 21–25, 27–29, 31–36] reported *Ascaris* (*A.*) *lumbricoides*, 18 papers [4, 9–12, 21–25, 27–29, 31, 33–36] reported hookworm, 12 papers [4, 10, 12, 21, 22, 24, 25, 27, 29, 31, 35, 36] reported *Strongyloides* (*S.*) *stercoralis*, 10 papers [4, 9, 12, 21, 23, 25, 29, 31, 33, 35] reported *Schistosoma* (*S.*) *mansoni*, 16 papers [4, 9, 11, 12, 21–29, 31, 35, 36] reported *Trichuris* (*T.*) *trichiura*, 7 papers [11, 22, 23, 25, 27, 29, 32] reported *Hymenolepis* (*H.) nana,* and 4 papers each reported *Taenia* species [22, 25, 27, 32] and *Enterobius* (*E.*) *vermicularis* [23–25, 29]. From this analysis, the pooled prevalence of *S. mansoni* (9.98%, (95%CI: 4.85, 15.10)) was the highest followed by *S. stercoralis* (7.74%, (95%CI: 4.24, 11.24)) and hookworm (6.91%, (95%CI: 4.22, 9.60)). The least pooled prevalence in the TB case group was *E.vermicularis* (Table 2).

**Table 2:** Pooled prevalence and odds ratio of each intestinal helminth among TB positive and TB negative participants in Africa and Asia

Helminths	Among TB positives		Among TB negatives		OR (95% CI)
	**No. of studies**	**PP (95% CI)**	**No. of studies**	**PP (95% CI)**	
***A. lumbricoides***	19	6.65 (4.90, 8.40)	11	6.62 (4.13, 9.12)	1.47 (0.83,2.59)
***Hookworm***	18	6.91 (4.22, 9.60)	12	6.58 (4.11, 9.06)	1.52 (0,91, 2.54)
***S. stercoralis***	12	7.74 (4.24, 11.24)	8	3.47 (1.44, 5.50)	2.67 (1.24, 5.76)^a^
***S. mansoni***	10	9.98 (4.85, 15.10)	7	8.05 (4.25, 11.84)	1.36 (0.92, 2.01)
***T. trichiura***	16	2.86 (1.68, 4.03)	10	3.00 (1.25, 4.76)	1.55 (0.71, 3.73)
***H. nana***	7	0.45 (0.09, 0.80)	4	0.49 (0.06, 0.91)	1.72 (0.45, 6.61)
***Taenia* species**	4	0.99 (0.18, 1.80)	4	0.47 (0.01, 0.92)	1.37 (0,28, 6.64)
***E. vermicularis***	4	0.37 (-0.07, 0.8)	4	0.98 (0.43, 1.54)	0.43 (0.73, 3.28)

*PP* Pooled prevalence, *CI* Confidence interval

^*a*^*significantly associated*

Regarding the TB negative (control) category, 11 articles [4, 9, 12, 21, 24, 28, 29, 33–36] reported *A. lumbricoides*, 12 articles [4, 9, 10, 12, 21, 28–30, 33–36] reported hookworm, 10 articles [4, 9, 12, 21, 24, 28, 29, 34–36] reported *T. trichiura*, 8 articles [4, 11, 21, 24, 29, 34–36] reported *S. stercoralis,* 7 articles [4, 9, 12, 21, 29, 30, 33] reported *S. mansoni*, and 4 articles each reported *H. nana* [12, 21, 29, 34]*, Taenia* species [12, 21, 29, 34] and *E. vermicularis *[4, 24, 29, 32]. Under this group of population the highest pooled prevalence was that of *S. mansoni* followed by *A. lumbricoides* (Table 2). Among all reported helminth types, only *S. stercoralis* (OR = 2.67 (95%CI 1.24, 5.76), *P* = 0.001, I^2^ = 72.9%) showed a statistically significant association to TB cases compared to TB negative participants (Table 2) (S3 file).

## Discussion

The overall pooled prevalence of intestinal helminth infection among TB patients was high (29.69%). In comparison to TB negative individuals, patients with TB were more prone to have intestinal helminth co-infection. This finding was in line with the review conducted in Ethiopia (33%) [15], and other individual reports in India (30.9%) [10] and Nigeria (28.1%) [36], but lower than the study conducted by Dessie et al. (36.1%) [37]. From the meta-regression analysis, unlike the previous review reported by Taghipour et al. [16], TB cases were approximately twice more at risk to have intestinal helminth infection than TB negative individuals (OR = 1.72 (95%CI: 1.20, 2.48)). Similar finding had been reported by Alemu et al. in Ethiopia [15]. By contrast, one study showed that early stage of helminth infection has a protective effect during subsequent *M. tuberculosis* infection [38].

Importantly, it is difficult to make conclusive chronological or causal relationships of the two agents, whether TB is a risk factor for helminth infection or vice versa. The immune modulation effect of intestinal helminths may have a prominent impact on the protective Th1 cell responses, which plays a major role for the development of cell-mediated immune responses during *M. tuberculosis* infection [1, 39]. Moreover, intestinal helminth-induced Th2 cell response may also lead to the up-regulation of regulatory T cells (Treg) that can down-modulate both Th1 and Th2 responses and interfere with their effector T-cell functions [40]. This action might be helpful for both *M. tuberculosis* and intestinal helminth coexistence and persistence.

In the subgroup analysis of included articles, the pooled prevalence of intestinal helminth co-infection was higher in Asia than Africa (35.30% vs 28.24%) (S2 file). However, the confidence intervals for both regions largely overlap. In addition, an individual paper from Malaysia, reported by Wong et al., showed the highest prevalence (85.4%) among all included papers. This may possibly have increased the pooled prevalence in the region. Furthermore, when we compare Ethiopia with other regions, the pooled prevalence of intestinal helminth co-infection was relatively lower (26.22%) than in other African countries (33.02%), yet not statistically significant (S2 file). Moreover, the current result (26.22%) is also less than the previous review conducted in Ethiopia (33%) [15]. This may be due to the fact that the previous review conducted in Ethiopia reported all types of intestinal parasites; including protozoan parasites, which are highly prevalent in the country. But in our case we specifically analyzed intestinal helminths. The difference of pooled prevalence across regions might be attributed to the small sample size and number of studies included in Africa and Asia for the analysis. In Asia, articles might be published in their local language that limits the number of eligible papers in the region, which could be considered as one of the limitations of this review. Furthermore, differences in the sensitivity of intestinal helminth diagnostic techniques might be considered as a reason for the variation.

In this systematic review and meta-analysis, different types of intestinal helminths were reported, with the leading parasites co-infected TB patients including *S. mansoni* followed by *S*. *stercoralis* and hookworm*.* The least pooled prevalence was found on *E vermicularis*. This may be due to application of inappropriate diagnostic modality. In case of *E. vermicularis* diagnosis, perianal tape impression is the preferable method, which most of the included articles did not apply. Moreover, this parasite is mostly prevalent at younger children than adult [41]. The pooled prevalence of *S. mansoni* was also highest among TB negatives. The above mentioned intestinal helminths are common in most areas of Africa and Asia. Nevertheless, except S*. stercoralis* (OR = 2.67 (95% CI 1.24, 6.76))*,* other individual parasites did not show statistically significant association with TB (Table 2) (S3 file). This finding was consistent with the previous review [16]. Different species of helminths infection may have distinct impact on susceptibility of the host for TB diseases. Some studies showed *S. stercoralis* exert a profound effect on the TB protective immune responses, which may increase susceptibility of the host to develop active TB [39, 42, 43].

In the current review, we have also examined some of the possible risk factors for intestinal helminth infection among TB patients. Hence, in the meta-regression analysis, a BMI < 18.5 kg/m^2^ was a significant factor associated with intestinal helminth infection among TB cases (OR = 2.75 (95%CI: 1.19, 6.38)). It is clear that malnutrition is associated with TB. Based on the finding in this review, the low BMIcan be considered as a coalescence factor for the co-infection of helminth and TB. Individual studies also support this finding [11, 22]. Nutritional effects (low BMI) could be one of the reasons for the susceptibility of the host [23, 44]. Another possible risk factor, HIV, was assessed and the pooled prevalence showed higher HIV co-infection among TB cases. However, it wasn’t statistically significant (OR = 0.88 (95%CI: 0.58, 1.52)). This finding was consistent with earlier reports [15, 20, 30]. In contrast, individual studies revealed a significant association of intestinal helminth and HIV infection among TB cases [24, 25]. Other risk factors like sex, age, educational status and residence did not show statistically significant association with helminth infection among TB cases. Similar findings have been reported with different individual studies [4, 11]. In contrast other studies reported that residence showed statistically significant association [22, 31].

Systematic review and meta-analysis, specifically addressing intestinal helminth and *M. tuberculosis* co-infection in Africa and Asia is very limited. As strength, the data can provide evidence about current status of intestinal helminth co-infection among TB cases in the developing world, where both infections pose a huge public health challenge. However, this review has a number of limitations. There was scarcity of studies in other African and Asian countries, in which the analysis may be suffering from small study effect. More than half of the studies (54%) included in this review were conducted in a single country, Ethiopia. The reason for small number of articles from Asia may be due to publication in their local language. Moreover, the search strategy missed unpublished articles at which publication bias might be very likely. In addition, since there is no ‘gold standard’ test for intestinal helminths detection, a variable range of diagnostic techniques applied in different studies could have increased the heterogeneity.

## Conclusions

In our review, we observed a high rate of concomitant tuberculosis and intestinal helminth infection with a considerable proportion. Among all helminthes, the coexistence of *S. stercoralis* and TB showed a statistically significant association, suggesting the need for further prospective epidemiological and mechanistic studies. In addition, low BMI was shown to have a statistically significant association with intestinal helminth infection among patients with TB. Hence, this study may suggest the need to screen intestinal helminth and to assess nutritional status for all patients presenting with TB infection. Finally, more studies on the epidemiology of TB and helminths co-infection with wider representation of different geographic regions of the global south, is needed to achieve a better understanding of the mutual interactions between both diseases.

### Supplementary Information

**Additional file 1: S1 Table. **The PRISMA check list of the review.

**Additional file 2: S2 Table. **Search strategy during articles review.

**Additional file 3: S3 Table. **Quality Assessment Tool for the Included Studies in the review.

**Additional file 4: S1 File. **The raw data extracted from eligible studies.

**Additional file 5: S2 File. **Subgroup analysis by region.

**Additional file 6: S3 File. **Odds ratio of different helminth types.

## Data Availability

All data pertaining to this study are contained and presented in this document and in the supplementary files.

## Abbreviations

BCG: Bacille Calmette-Guerin
BMI: Body Mass Index
CI: Confidence Interval
HIV: Human Immunodeficiency Virus
LTB: Latent TB
*M. tuberculosis*: *Mycobacterium tuberculosis*
MeSH: Medical Subject Headings
OR: Odds Ratio
PRISMA: Systematic Reviews and Meta-Analyses
PRR: Pattern Recognition Receptors
STH: Soil Transmited Helminth
TB: Tuberculosis
WHO: World Health Organization
