# Supplementary material for: Intestinal helminth co-infection and associated factors among pulmonary tuberculosis patients in Africa and Asia: a systematic review and meta-analysis
Source: BMC Infect Dis. 2023 Oct 30;23:739. doi: 10.1186/s12879-023-08716-9 (PMC10614413; doi:10.1186/s12879-023-08716-9)
Supplement: Supplementary file 1 — Additional file 1: S1 Table. The PRISMA check list of the review. [file 12879_2023_8716_MOESM1_ESM.doc]

| **Section/topic** | **#** | **Checklist item** | **Reported on page #** |
| --- | --- | --- | --- |
| **TITLE** | | |  |
| Title | 1 | **Intestinal helminth co-infection and associated factors among pulmonary tuberculosis patients in Africa and Asia: A systematic review and meta-analyses** | 1 |
| **ABSTRACT** | | |  |
| Structured summary | 2 | ***Background*:** Tuberculosis (TB) has huge public health importance, and intestinal helminths are also prevailing in the same areas where TB is rampant. Data about the burden of intestinal helminth and TB co-infection in these areas are fragmented. In this systematic review and meta-analysis we compile the current literatures and generate pooled prevalence. We also identity factors associated with intestinal helminth co-infection among TB patients.  ***Methods*:** Original articles published in English language up to March 23, 2022 were systematically searched from electronic database (PubMed/Medline, Scopus, Science Direct, Google Scholars and HINARI). The search was done using medical subject heading terms and keywords. Identified articles were exported into the EndNote library. The identified articles were screened using PRISMA flow diagram. Then the methodological quality of included articles was evaluated and rated using the modified version of Newcastle-Ottawa Scale. Data were extracted using Microsoft Excel. Sensitivity analysis and Egger regression test were used for the assessment of heterogeneity and publication bias. Finally the results are presented with a meta-analysis of pooled estimates, forest plots, and tables. The quantitative data were analyzed using Stata version 14.  ***Results*:** From a total of 5457 searched articles, 22 eligible articles were included in the review. The pooled prevalence of helminth co-infection among TB cases was 29.69% (95%CI: 21.10, 38.29). TB patients were found to more frequently harbor one or more intestinal helminth than TB negative individuals (OR= 1.72 (95%CI: 1.20, 2.48)*.* Among the reported helminths, *Schistosoma mansoni* and *Strongyloides* *stercoralis* had the highest pooled prevalence among TB cases. However, unlike other individual helminths, only *Strongyloides* *stercoralis* (OR, 2.67; 95% CI, 1.20–6.76) had significant association with TB cases compared to TB negatives. BMI was significantly associated with intestinal helminth co-infection among TB patients (OR=2.75, (95%CI: 1.19, 6.38)).  ***Conclusions*:** About one third of TB patients were co-infected with one or more intestinal helminths and they were more likely to have intestinal helminth than their TB negative counter parts. The higher prevalence of helminth infection in TB cases might indicate that co-infection promotes active TB disease. Thus, routine intestinal helminth screening and assessment of their nutritional status is recommended for TB patients. | 2-3 |
| **INTRODUCTION** | | |  |
| Rationale | 3 | Tuberculosis (TB) and intestinal helminth infections are geographically co-existing health problems and the most prevalent infectious diseases, mainly in middle and low-income countries (1). Helminth infection is an area which remains largely ignored. Globally, more than 2 billion people are infected with intestinal helminths, and particularly soil transmitted helminths (STHs) affect more than 1.5 billion people in Africa, Asia and Latin America (2). About one third of the people worldwide are also infected with *Mycobacterium (M.) tuberculosis* (1). Africa and South-East Asia account for more than 70% of the global TB burden (3). The geographical overlap of TB and other debilitating infectious diseases, such as intestinal helminths and HIV, makes the situation worse than in other WHO regions (4). Moreover, intestinal helminth infection and TB are considered as poverty related diseases. Hence, the co-infection of intestinal helminths and *M. tuberculosis* is common, especially in African and Asian countries, where the burden of poverty related factors is high (3, 5, 6). Up on this information, our review is specifically targeted in Africa and Asia to compile and analyze available evidences related to *M. tuberculosis* and intestinal helminths co-infection.  Co-infection from helminth and *M. tuberculosis* is not surprising in geographical areas where both diseases are frequent. The two infectious pathogens use several self-governing mechanisms to affect immune responses of the host and impact on their infection outcomes (7, 8). Thus, it is conceivable that both infections can mutually change the susceptibility to and the course of disease. During *M. tuberculosis* infection the immune response of the host is polarized to the Th1 type of immune response. Hence, the Th2 type of immune response, which is important to combat helminth infection, will be down-regulated with an antagonist effect of cell mediated immunity. Moreover, this could be caused by innate immune training, altered barrier function, and nutritional effects (low BMI) (9, 10).  Given this potential immunological interference with host defense to TB by underlying helminth infection, and vice versa, the question for epidemiological evidence of association of both infections has been asked in several studies. Different individual studies showed significant correlation between intestinal helminth and TB (11-14). By contrast, one study showed that early stage of helminth infection has a protective effect during subsequent *M. tuberculosis* infection (15). Other studies did not find an association (16, 17). One review of 11 studies done in a single country (Ethiopia) showed higher pooled prevalence of intestinal parasite infection among TB cases (18). One more single review of 20 studies at the global scale, reported by Taghipour *et al,*  also showed considerable pooled prevalence of intestinal helminth infection (19). The above two reviews indicated substantial rate of helminth infection among TB cases. However, many questions remain to be answered, such as regional subgroup analysis for differences between geographic regions, or the role of possible risk factors associated with co-infection. In addition, the study of co-infections with helminths and TB is an active research field requiring intermittent analyses of the current state of the literature to obtain up-to-date information, especially in regions of low/middle-income countries.  Hence, this systematic review and meta-analysis is aimed to generate up-to-date information about the burden of intestinal helminth and *M. tuberculosis* co-infections in African and Asian countries. Moreover, the subgroup analysis of intestinal helminth co-infection across different regions/countries and assessment of possible risk factors may give a more detailed picture to comprehend the effect of helminths infection on active TB development or the effect in vice-versa. | 2-5 |
| Objectives | 4 | The current review was planned to generate updated compiled data about the burden of intestinal helminth and *M .tuberculosis* co-infections in Asian and African countries. Moreover, the subgroup analysis of intestinal helminth co-infection across different regions and countries may give a clear picture to comprehend the problem and take evidence based measures. | 5 |
| **METHODS** | | |  |
| Protocol and registration | 5 | The protocol of this review was developed based on the Preferred Reporting Items for Systematic Reviews and Meta-Analyses (PRISMA) reporting checklist and registered at Prospective Register of Systematic Reviews (PROSPERO) ID: CRD42022315731. | 5 |
| Eligibility criteria | 6 | Freely accessible original articles (prospective and retrospective cross-sectional, case-control, and cohort studies), which are reported on tuberculosis and intestinal helminths co-infection in different countries of Asia and Africa, and written in English language were included. However, review papers, conference papers, editorials, commentaries, case reports/case series, and articles published out of the study population and English language were excluded. Studies were reviewed based on the criteria of PICOS (participants, interventions, comparison, outcome, and study setting). | 5 |
| Information sources | 7 | Original articles published in English up to March/2022 were systematically searched from the accessible electronic databases such as, PubMed, Scopus, Science Direct, Google Scholars and HINARI. Grey literatures were retrieved from university databases and article preprint sources, medRxiv and bioRxiv. The reference lists of related reviews were screened to identify additional articles. | 6 |
| Search | 8 | The article search was done using medical subject headings (MeSH) terms and keywords with an appropriate combination using Boolean operators “AND” and “OR”. The search algorithm was “((((("Helminths"[Mesh]) OR "Intestinal Diseases, Parasitic"[Mesh]) AND "Mycobacterium tuberculosis"[Mesh]) OR "Tuberculosis, Pulmonary"[Mesh]) AND "Coinfection"[Mesh])”, (**S1 file**). | 6 |
| Study selection | 9 | All of the identified articles were exported into the EndNote library. After removing the duplicate, articles identified as potentially relevant during title and summary screening were further evaluated by reading the full work of the paper. Article selection was done following the PRISMA flow chart (Error: Reference source not found). Papers which were not eligible for the review were excluded and the reason for exclusion is documented in the flow chart. | 6 |
| Data collection process | 10 | Data extractions were carried out after piloting the data extraction sheet. Data were extracted by two of the authors (YZ, DM) independently and any discrepancies on the data item were resolved by discussion and 3^rd^ party judgment (BT). | 6 |
| Data items | 11 | After the selection of eligible articles, the findings of the papers were extracted using a data extraction template. The data extraction included, the name of the first author, year of publication, study area/country, study design, sample size, mean age, TB screening method, intestinal helminth screening method, and the number and types of intestinal helminthes. Subgroup data containing outcomes of intestinal helminths among TB patients and TB negative participants, multiple helminthic infections, sex, HIV status of TB patients, and BMI were extracted in Microsoft excel (**S2 file**). | 8 |
| Risk of bias in individual studies | 12 | To assess risk of bias, two authors independently used the modified Newcastle Ottawa Scale for risk of bias assessment tool. Each item scored one point and discrepancies were resolved by the third reviewer. | 9 |
| Summary measures | 13 | Transformed pooled prevalence and determinants with 95% CI was the summary measure used. | 9 |
| Synthesis of results | 14 | The collected data were analyzed using qualitative and quantitative measures via Stata 14 statistical software. We were computed the effect size (ES) of the prevalence and the determinants of helminth co-infections in TB patients.. Forest plot was used to assess the effect of risk factors. In the forest plot, the box indicated weight of articles from random effect analysis. The crossed line is the 95% confidence interval (CI), the solid vertical line is zero to x-axis. | 9 |

Page 1 of 2

| **Section/topic** | **#** | **Checklist item** | **Reported on page #** |
| --- | --- | --- | --- |
| Risk of bias across studies | 15 | Sensitivity analysis and Egger regression test were used for the assessment of heterogeneity and publication bias. The I^2^ statistic value of 25%, 50%, and 75% were used to declare the heterogeneity test as a low, medium, and high, respectively. In case of significant heterogeneity, a random effect model was used for the analysis (43). The fixed model effect was also used for the analysis of non-significant heterogeneity. Publication bias was explored using visual inspection of the funnel plot (Error: Reference source not found**)** | 9 |
| Additional analyses | 16 | We carried out a subgroup analysis and meta-regression of heterogeneous papers, with the relationship between helminthic infection and tuberculosis, according to the region of the study conducted. | 10 |
| **RESULTS** | | |  |
| Study selection | 17 | A total of 5456 research articles were explored from different open-access scientific data bases, registers and other sources, as explained from the methodology part. Among these, 5160 papers were removed due to duplication, irrelevance to our objective and other reasons. Only 296 research articles were screened by their title and abstract. Finally 46 research articles were selected for full paper evaluation | 10 |
| Study characteristics | 18 | After intensive screening, we found 22 studies (9, 11-14, 23-39) as eligible articles for final analysis (Error: Reference source not found). From the included articles the number of case control, cohort and cross-sectional studies were 6, 1 and 15, respectively (Error: Reference source not foundError: Reference source not found). The total number of participants among TB cases was 4,986 and those of the control group 3,246 with a sample size ranging from 16 to 668. The mean age of participants among TB cases and TB negatives (controls), were 35.27±7.30 years and 35.75±8.71 years, respectively. | 12 |
| Risk of bias within studies | 19 | The quality of each study were rated using the following scoring algorithms: points of ≥7, 3 to 6 and <3 were considered as “good”, “fair”, and “poor” quality studies, respectively (Error: Reference source not found **file**). Therefore, in order to improve the validity of this systematic review result, we only included primary studies with fair and good quality (Error: Reference source not found) (44).  From the analysis, Egger’s test showed a publication bias (P=0.016) and the funnel plot was almost asymmetrical (Error: Reference source not found). | 9,10 |
| Results of individual studies | 20 | The overall pooled prevalence of intestinal helminth infection among TB patients reported from 22 selected articles was 29.69% (95%CI: 21.10, 38.29) (Error: Reference source not found**).** Among the selected articles, 15 reported both intestinal helminth infection among TB cases and TB negative participants. Thus, the pooled prevalence of intestinal helminth infection among TB cases and controls were 30.40% ((95%CI: 17.75, 43.05) P<0.001, I^2^=98.9.0%) and 21.65%, ((95%CI: 13.78, 29.51), P<0.001, I^2^=98.6%), respectively (Error: Reference source not found **andError: Reference source not found**). A wider range in the prevalence of intestinal helminth co-infection among TB patients (1.86% in Iran and 85.37% in Malaysia) and TB negatives was observed (0.55% in Iran and 89.09% in Malaysia) (Error: Reference source not found) ([S1 File](../../../../E:%5CCLRs%5CReview%5CTB%20and%20Helminth%5CHelminth%20and%20TB%20review%202022%5CNew%20Versions%5CDocuments%5CHelminth%20TB%20coinfection_YZ_August_11_2022%5CSuplmentary%5CS1%20File-%20The%20raw%20data%20extracted%20from%20eligible%20studies.xlsx)). | 16 |
| Synthesis of results | 21 | In this review we had estimated the pooled prevalence of helminth co-infection at 95% CI in African and Asian countries. In addition we had computed the risk factors of the co-infection trough estimating the odds ratio. And presented in forest plot from figure 3-8. | 11-16 |
| Risk of bias across studies | 22 | Funnel plot and sensitivity tests were used to assess the risk of bias of the included studies. | 14 |
| Additional analysis | 23 | Determinant factor analysis was done | 12-16 |
| **DISCUSSION** | | |  |
| Summary of evidence | 24 | *M .tuberculosis* and intestinal helminths are two substantial causative agents for morbidity and mortality in low and middle income countries, and TB and intestinal helminth infections geographically overlap (2, 40). Even though decades of intensive efforts have been made, TB continues to be a significant health problem in the developing world. The majority of global TB burden is accounted in the continent of Africa and Asia (3). Compiled data about the association of intestinal helminth and TB in these continents should not be overlooked. This systematic review and meta-analysis was conducted to determine the pooled prevalence and associated factors with intestinal helminth infection among TB patients in Africa and Asia. After exhaustive search, 22 eligible articles (9, 11-14, 23-39) were retrieved and included for final analysis. | 23-26 |
| Limitations | 25 | The overall pooled prevalence of intestinal helminth infection among TB patients was high (29.69%). In comparison to TB negative individuals, patients with TB were more prone to have intestinal helminth co-infection. This finding was in line with the review conducted in Ethiopia (33%) (18) and other individual reports in India (30.9%) (12) and Nigeria (28.1%) (39), but lower than the study conducted by Dessie *et al* (36.1%) (41). From the meta-regression analysis, unlike the previous review reported by Taghipour *et al* (19), TB cases were approximately twice more at risk to have intestinal helminth infection than TB negative individuals, OR=1.72, (95%CI: 1.20, 2.48). Similar finding had been reported by Alemu *et al* in Ethiopia (18). Importantly, it is difficult to make conclusive chronological or causal relationships of the two agents, whether TB is a risk factor for helminth infection or vice versa. The immune modulation effect of intestinal helminths may have a prominent impact on the protective Th1 cell responses, which plays a major role for the development of cell-mediated immune responses during *M. tuberculosis* infection (1, 42). Moreover, intestinal helminth-induced Th2 cell response may also lead to the up-regulation of regulatory T cells (Treg) that can down-modulate both Th1 and Th2 responses and interfere with their effector T-cell functions (43). This action might be helpful for both *M. tuberculosis* and intestinal helminth coexistence and persistence. | 16 |
| Conclusions | 26 | Conferring on the data compiled in this review, the prevalence of *M. tuberculosis* and intestinal helminth co-infection remains high, and TB patients were more likely to have one or more intestinal helminth infection. Accordingly, we conclude that there is a strong relationship between TB and intestinal helminth infection. In fact, the significance of an association may be varying in between different intestinal parasites. In this meta-analysis, *S. stercoralis* infection was the only helminth infection which showed statistically significant association with active TB, suggesting the need for further epidemiological and mechanistic studies. From the analyzed host determinant factors, only BMI was significantly associated with intestinal helminth infection among TB cases. Hence, routine intestinal helminth screening and assessment of their nutritional status is recommended for all TB patients. Finally, more studies on the epidemiology of co-infection with TB and helminths are needed, including a better and wider representation of different geographic regions of the global South, to achieve a better understanding of the mutual interactions between both diseases. | 26 and 27 |
| **FUNDING** | | |  |
| Funding | 27 | This work was supported by funding from Deutsche Forschungsgemeinschaft (DFG) (grant number LA 1262/8-1). | 27 |
