# Supplementary material for: Intestinal helminth co-infection and associated factors among pulmonary tuberculosis patients in Africa and Asia: a systematic review and meta-analysis
Source: BMC Infect Dis. 2023 Oct 30;23:739. doi: 10.1186/s12879-023-08716-9 (PMC10614413; doi:10.1186/s12879-023-08716-9)
Supplement: Supplementary file 2 — Additional file 2: S2 Table. Search strategy during articles review. [file 12879_2023_8716_MOESM2_ESM.docx]

**S2 Table**

| **No** | **MeSH term** | **Database** | **No. of articles** | **Selected by title** | **Search date(mm/dd/yyyy)** |
| --- | --- | --- | --- | --- | --- |
| 1 | (((((((("Helminths"[Mesh]) OR "Helminthiasis"[Mesh]) OR "Intestinal Diseases, Parasitic"[Mesh]) AND "Mycobacterium tuberculosis"[Mesh]) OR "Tuberculosis, Pulmonary"[Mesh]) OR "Tuberculosis"[Mesh]) AND "Africa"[Mesh]) OR "Asia"[Mesh]) AND "Coinfection"[Mesh] | **PubMed** | **2101** | **23** | **03/11/2022** |
| **3** | (((("Mycobacterium tuberculosis"[Mesh]) AND "Intestinal Diseases, Parasitic"[Mesh]) OR "Helminths"[Mesh]) AND "Coinfection"[Mesh]) | **PubMed** | **122** | **4** | **03/12/2022** |
| **4** | Helminth co-infection and associated factors among tuberculosis patients in Africa | **Hinari** | **286** | **8** | **03/15/2014** |
| **5** | Helminth coinfection and associated factors among tuberculosis patients in Africa or Asia | **Google scholars** | **2546** | **58** | **03/14/2022** |
| **6** | Helminth co-infection and associated factors among tuberculosis patients in Africa | **Science Direct** | **147** |  | **03/14/2022** |
|  | ( TITLE-ABS-KEY ( helminths )  OR  TITLE-ABS-KEY ( "Intestinal parasite" )  OR  TITLE-ABS-KEY ( "Intestinal parasitic infection" )  AND  TITLE-ABS-KEY ( "Mycobacterium tuberculosis" )  OR  TITLE-ABS-KEY ( tuberculosis ) )  AND  ( LIMIT-TO ( DOCTYPE ,  "ar" ) )  AND  ( LIMIT-TO ( LANGUAGE ,  "English" ) ) | Scopus | **357** | **65** | **03/23/2022** |
|  | ( TITLE-ABS-KEY ( helminths )  OR  TITLE-ABS-KEY ( "Intestinal parasite" )  OR  TITLE-ABS-KEY ( "Intestinal parasitic infection" )  AND  TITLE-ABS-KEY ( "Mycobacterium tuberculosis" )  OR  TITLE-ABS-KEY ( tuberculosis )  AND  TITLE-ABS-KEY ( "Co-infection" )  OR  TITLE-ABS-KEY ( "coinfection" ) )  AND  ( LIMIT-TO ( DOCTYPE ,  "ar" ) )  AND  ( LIMIT-TO ( LANGUAGE ,  "English" ) ) | Scopus | **95** | **23** | **03/23/2022** |
