# Supplementary material for: Intestinal helminth co-infection and associated factors among pulmonary tuberculosis patients in Africa and Asia: a systematic review and meta-analysis
Source: BMC Infect Dis. 2023 Oct 30;23:739. doi: 10.1186/s12879-023-08716-9 (PMC10614413; doi:10.1186/s12879-023-08716-9)
Supplement: Supplementary file 6 — Additional file 6: S3 File. Odds ratio of different helminth types. [file 12879_2023_8716_MOESM6_ESM.docx]

**S3 file**
